# ANTI-AGING COMPOUNDS METFORMIN AND THIOFLAVIN T CAN ACT ON GENETICALLY DIVERSE, PROTEOSTASIS-COMPROMISED STRAINS

**DOI:** 10.1093/geroni/igac059.2908

**Published:** 2022-12-20

**Authors:** Monica Driscoll, Brian Onken, Yunpeng Xu, Yuhua Song, Madhuri Achanta, Christine Sedore, Patrick Phillips, Gordon Lithgow

**Affiliations:** Rutgers, The State University of New Jersey, New Brunswick, New Jersey, United States; Rutgers University, Piscataway, New Jersey, United States; Rutgers University, Piscataway, New Jersey, United States; Rutgers University, Piscataway, New Jersey, United States; Rutgers University, Piscataway, New Jersey, United States; University of Oregon, Eugene, Oregon, United States; University of Oregon, Eugene, Oregon, United States; Buck Institute, Novato, California, United States

## Abstract

The Caenorhabditis Intervention Testing Program (CITP) seeks to identify robust and reproducible pro-longevity interventions affecting a genetically diverse panel of representative Caenorhabditis strains from each of 3 species – C. elegans, C. briggsae, and C. tropicalis. The CITP test strains represent genetic diversity on the order of mouse to human. Anchoring principles of the CITP are that efficacious interventions that target fundamental and conserved biology will hold strong potential for translation to higher organism benefit and that such interventions can be rapidly identified using Caenorhabditis models. The CITP is creating multi-species “at risk” test sets, in which components of aging hallmarks (such as proteostasis, stress response circuits) are genetically compromised in diverse genetic backgrounds. Such genetic test sets might expand understanding of intervention action and help rank compounds for translational testing. Toward this end, we used CRISPR to generate deletion mutants of arsenite-inducible protein (C. elegans aip-1, human AIRAP/ARAPL) in which proteostasis and oxidative stress responses are compromised across phyla. All aip-1 mutants are short lived compared to their control wild type strains, highlighting the essential role of proteostasis in maintaining healthy aging. Our previous studies identified metabolic modulator Metformin and anti-amyloid Thioflavin T as effective pro-longevity, pro-healthspan interventions in multiple CITP test strains. We find that Metformin and Thioflavin T impact the aip-1 deletion strains similarly to WT variants. Together, our results demonstrate that Metformin and Thioflavin-T can positively impact lifespan in diverse genetic backgrounds in which proteostasis is compromised, suggesting wide-ranging health benefits of these potent pro-longevity compounds.

